# Mutations in EPAS1 in congenital heart disease in Tibetans

**DOI:** 10.1042/BSR20181389

**Published:** 2018-12-18

**Authors:** Hong Pan, Qiuhong Chen, Shenggui Qi, Tengyan Li, Beihong Liu, Shiming Liu, Xu Ma, Binbin Wang

**Affiliations:** 1 Center for Genetics, National Research Institute for Family Planning, Beijing 100081, China; 2 Graduate School of Peking Union Medical College, Beijing, China; 3 Qinghai High Altitude Medical Research Institute, No.7, Zhuanchang Road, West District, Xining 810012, China

**Keywords:** Congenital heart disease, EPSA1 gene, hypoxia, mutation, Tibetans

## Abstract

*EPAS1* encodes HIF2 and is closely related to high altitude chronic hypoxia. Mutations in the *EPAS1* coding sequence are associated with several kinds of human diseases, including syndromic congenital heart disease (CHD). However, whether there are rare *EPAS1* coding variants related to Tibetan non-syndromic CHD have not been fully investigated. A group of 286 Tibetan patients with non-syndromic CHD and 250 unrelated Tibetan healthy controls were recruited from Qinghai, China. Sanger sequencing was performed to identify variations in the *EPAS1* coding sequence. The novelty of identified variants was confirmed by the examination of 1000G and ExAC databases. Control samples were screened to establish that the rare candidate variants were specific to the Tibetan patients with non-syndromic CHD. Bioinformatics software was used to assess the conservation of the mutations and to predict their effects. The effect of *EPAS1* mutations on the transcription of its target gene, *VEGF*, was assessed by dual-luciferase reporter assay. The mammalian two-hybrid assay was used to study the protein interactions between HIF2 and PHD2 or pVHL. We identified two novel *EPAS1* mutations (NM_001430: c.607A>C, p.N203H; c.2170G>T, p.G724W) in two patients. The N203H mutation significantly affected the transcription activity of the *VEGF* promoter, especially in conditions of hypoxia. The N203H mutation also showed enhanced protein–protein interactions between HIF2 and PHD2, and HIF2 and pVHL, especially in conditions of hypoxia. However, the G724W mutation did not demonstrate the same effects. Our results indicate that EPAS1 mutations might have a potential causative effect on the development of Tibetan non-syndromic CHD.

## Introduction

Congenital heart disease (CHD) is a problem with the structure and function of the heart that is present at birth. CHD affects ∼1% of live births and remains the leading cause of mortality from birth defects [1]. Most CHD patients have isolated heart defects and are referred to as non-syndromic. Although many genes have been identified as causing rare inherited forms of CHD, the causes of most cases of sporadic CHD remain unknown [2]. Many reports indicate that genetic changes in the hypoxia-inducible factors (HIFs) pathway are linked to human adaptation to high altitude, especially in Tibetans [3]. Our previous study showed that the rate of CHD in Tibetan children increased significantly with increasing altitude levels, suggesting that CHD in Tibetan children may be associated with altitude levels, as well as with hypoxia [4].

**Table 1:** Clinical information and bioinformatics prediction results

EPAS1 mutation (NM_001430 )		No. of cases	Phenotype	Gender (M/F)	Ethnic group	Age at diagnosis (year)	Frequency		Conservative (Y/N)	Bioinformatics prediction		
DNA changes	AA changes						ExAC	1000G		Mutation Taster	PolyPhen-2	SIFT
c.607A>C	p.N203H	1	PDA	F	Tibetan	13	0	0	Y	Disease causing	Probably damaging	Damaging
c.2170G>T	p.G724W	1	VSD	M	Tibetan	15	2	0	Y	Disease causing	Probably damaging	Damaging

Under hypoxic conditions, HIFs pathways are activated and function as oxygen-sensing mechanisms to adapt to the chronic low-oxygen conditions [5]. HIF2 is encoded by endothelial PAS domain protein 1 (*EPAS1*) and plays an important role in the HIFs pathway. In normoxia, EPAS1 can be hydroxylated by prolyl-hydroxylase domain 2 (PHD2) and then bound to von Hippel–Lindau tumor suppressor (VHL) protein, causing rapid ubiquitination and degradation [6,7]. However, in hypoxia, this process can be inhibited [8]. *EPAS1* variations are related to plateau adaption and altitude-related diseases [9–14]. Recently, *EPAS1* gain-of-function somatic mutations were identified in pheochromocytomas and paragangliomas in patients with syndromic CHD [15]. *EPAS1* affects vascular endothelial growth factor (*VEGF*) expression, which is involved in angiogenesis and plays an essential role in human heart development [16–18]. Thus, we speculate that *EPAS1* also acts on the early development of the human heart.

However, whether there are rare *EPAS1* coding region variants related to Tibetan non-syndromic CHD remain to be established. The present study is to identify *EPAS1* mutations in Tibetan patients with non-syndromic CHD, and these results might help us improve our understanding of the genetic causes of non-syndromic CHD in the plateau hypoxic environment.

## Materials and methods

### Subjects

The study population comprised 286 Tibetan patients with CHD who were recruited from the Cardiovascular and Cerebrovascular Disease Hospital of Qinghai Province, China (female: 149, average age: 16.69 ± 14.03 years; male: 137, average age: 14.34 ± 13.68 years). All patients with CHD were diagnosed and classified according to the CHD guidelines issued by the American College of Cardiology/American Heart Association. Patients with abnormal karyotype or other combined symptoms were excluded. All cases were confirmed by surgery and/or cardiac catheterization and/or color Doppler echocardiography. All patients were diagnosed with non-syndromic CHD, including atrial septal defect, patent ductus arteriosus (PDA) and ventricular septal defect (VSD) (Supplementary Table S1). Unrelated, healthy, sex- and age-matched Tibetan health samples from Qinghai Province were selected as controls (*n*=250). All participants are Tibetans who have lived in the Qinghai-Tibet Plateau for generations. The present study was approved by the Medical Ethics Committee of the Cardiovascular and Cerebrovascular Disease Hospital of Qinghai Province, and written informed consent was obtained from all subjects and/or guardians.

### Mutational analysis and bioinformatics

Genomic DNA was extracted from peripheral blood leukocytes using the HiPure Blood DNA Mini Kit (Qiagen, Hilden, Germany) following the manufacturer’s protocol. The human *EPAS1* gene (NM_001430.4) is located on 2p21 and contains 17 exons. All human *EPAS1* coding regions were amplified by polymerase chain reaction (PCR) in 15 reactions. The PCR products were sequenced by Sanger sequencing (BGI-Huada, Shenzhen, China). The novelty of all identified variants was determined by examination of 1000 Genomes (1000G) and Exome Variant Server (ExAC) databases. Sanger sequencing was used to confirm that rare candidate variants were not also present in the healthy Tibetan controls. The effect of the mutations on protein structure and function was predicted using Mutationtaster, PolyPhen-2, and SIFT. Conservation analysis was performed using CLC Main Workbench Software (Aarhus, Denmark). Primers used for PCR and sequencing are presented in Supplementary Table S1.

## Plasmid construction

The open reading frame (ORF) of *EPAS1* was amplified by PCR from human cell line cDNA and inserted into the pcDNA3.1(+) vector (Invitrogen, Carlsbad, CA, U.S.A.) to create the pcDNA3.1(+)-EPAS1 expression plasmid. *EPAS1* mutations were constructed using the Quick Change Lightning Site-Directed Mutagenesis kit (Stratagene, La Jolla, CA, U.S.A.). The introduced mutations were confirmed by Sanger sequencing. The *VEGF* promoter was amplified by PCR from human genomic DNA and cloned into the pLG3-basic luciferase reporter vector. Wild-type (WT) and mutant (MUT) *EPAS1* fragments were amplified by PCR, using pcDNA3.1(+)-EPAS1 (WT and MUT) as template DNA and inserted into the pFN11A (BIND) Flexi^®^ Vector (Promega, Madison, WI, U.S.A.). The ORF of VHL was also amplified by PCR from human cell line cDNA and inserted into the pFN10A (ACT) Flexi^®^ Vector (Promega). All primers used are listed in Supplementary Table S2.

### Cell culture and transient transfection

The AC16 human cardiomyocyte cell line was maintained in Dulbecco’s Modified Eagle Medium supplemented with 10% fetal bovine serum, 100 mg/ml penicillin and 100 mg/ml streptomycin in a humidified atmosphere containing 5% CO_2_ at 37°C. Transfection was performed using Lipofectamine 3000 (Invitrogen Corporation, Carlsbad, CA, U.S.A.). A hypoxia-mimetic agent, deferoxamine (DFO; Sigma-Aldrich), was added to the culture medium at a concentration of 50 µM and incubated for 24 h to simulate a hypoxic environment [19].

### Reverse transcription-quantitative polymerase chain reaction (RT-qPCR)

AC16 cell total RNA was extracted with TRIzol reagent 24 h post-transfection as per manufacturer’s instructions (Invitrogen, Carlsbad, CA, U.S.A.). cDNA was synthesized for RT-qPCR using a high-capacity cDNA reverse transcription kit (Applied Biosystems, Foster City, CA, U.S.A.) using total RNA as the template as per manufacturer’s instructions. RT-qPCR was performed using the StepOne Real-Time PCR System and SYBR Green dyes (Takara, Otsu, Shiga, Japan) according to the manufacturer’s protocol. Relative quantification was performed using the 2^−△△*C*^_t_ method, with β-actin as the endogenous control. RT-qPCR primers used are listed in Supplementary Table S2.

### Dual-luciferase reporter gene assay and mammalian two-hybrid assay

The dual-luciferase reporter assay system (Promega) was used to study the effect of *EPAS1* on the transcription of its target gene, *VEGF*. The Renilla luciferase control pREP7-RLu, pcDNA3.1-EPAS1 (WT or MUT), and pGL3basic-VEGF promoter plasmids were co-transfected into AC16 cells. Cells were lysed 24 h post-transfection and luciferase activity measured according to the manufacturer’s instructions. In addition, the CheckMate™/Flexi^®^ Vector System (Promega) was used to study interactions between HIF2 and PHD2 or pVHL proteins. Three plasmids, pFN11A(BIND)-EPAS1 (WT or MUT), pFN10A(ACT)-PHD2 and –VHL, and the pGL4.31 [luc2P/GAL4UAS/Hygro] vector were cotransfected into AC16 cells. Twenty-four hours after transfection, cells were lysed and measured in the same way as mentioned above.

## Statistical analysis

The results are presented as the mean of three independent experiments performed in triplicate, and the error bars denote the standard deviation. The independent samples *t*test was adopted to determine statistical significance of unpaired samples. **,*P*<0.01; ***,*P*<0.001 vs. empty vector. ^#^,*P*<0.05; ^##^,*P*<0.01; ^###^,*P*<0.001 vs. wild-type construct. All data were analyzed by Prism Demo 5 software (GraphPad Software Inc., La Jolla, CA, U.S.A.).

## Results

### Two novel *EPAS1* mutations were identified in Tibetan non-syndromic CHD patients

**Figure 1:**
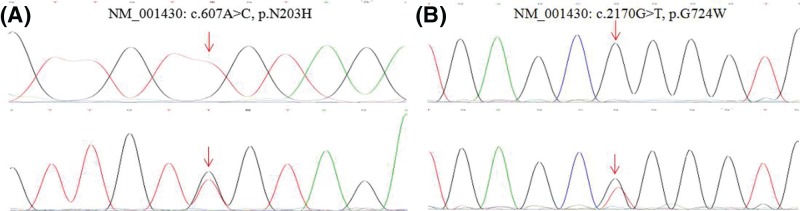
Electropherogram of the HIF2 N203H and G724W mutations (**A**) The upper peak shows the control and the lower peak shows the N203H mutation. (**B**) The upper peak shows the control and the lower peak shows the G724W mutation.

We identified two novel *EPAS1* mutations (NM_001430: c.607A>C, p.N203H; c.2170G>T, p.G724W) from a total of 286 Tibetan patients from Qinghai Province, China, with non-syndromic CHD (Figure 1, Table 1). The two subjects carrying the mutations are Tibetan residents and both had always lived at an altitude of over 2000 m. The N203H mutation was identified in a 13-year-old girl who had been diagnosed with PDA. This mutation was not in 1000G and ExAC databases. The G724W mutation was identified in a 15-year-old boy who had been diagnosed with VSD. This mutation had been reported twice in the ExAC database. Both N203H and G724W mutations were not detected in the control group. The amino acid sequences affected by these mutations are highly conserved across several species. Mutations in these regions may affect protein structure and function, as predicted by Mutationtaster, PolyPhen-2, and SIFT analyses.

### N203H mutation enhanced *VEGF* transcription

**Figure 2:**
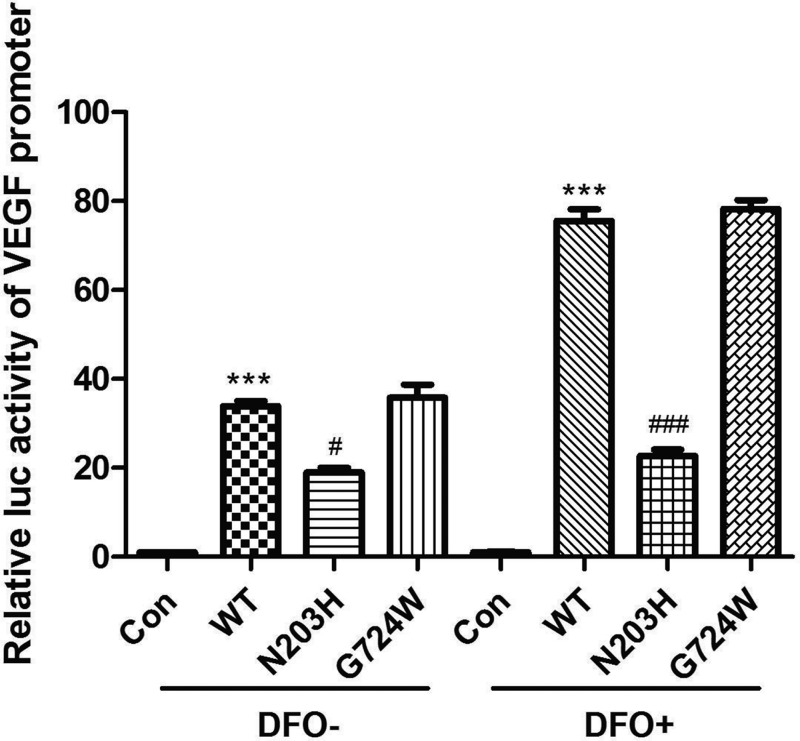
Effect of *EPAS1* mutations on *VEGF* transcription The dual-luciferase assay was performed to evaluate *VEGF* promoter activity. Cells were grown in conditions of normoxia (DFO-) or hypoxia (DFO+) for 24 h. The HIF2 N203H mutation significantly affected HIF2-mediated *VEGF* transcription, especially in conditions of hypoxia. ***p<0.001 vs. empty vector; #*p*<0.05; ###*p*<0.001 vs. wild-type (Student’s t-test).

The dual-luciferase assay was used to evaluate whether the identified *EPAS1* mutations affected *VEGF* transcription. In conditions of normoxia or hypoxia for 24 h, cells transfected with the wild-type construct produced significantly more luciferase activity than did those transfected with the pcDNA3.1(+) empty vector (***P<0.001 and ***P<0.001). There was no difference in the luciferase activity of cells transfected with the wild-type and G724W mutation constructs. However, cells transfected with the N203H mutation construct showed a significant decrease in luciferase activity compared with that observed in cells transfected with the wild-type construct (^#^*P*<0.05 and ^###^*P*<0.001). In hypoxic conditions, cells transfected with the wild-type construct exhibited approximately twice the luciferase activity than they did in normoxic conditions. The luciferase activity of cells transfected with the N203H mutation construct was about half and about one-third of that of cells transfected with the wild-type construct grown in conditions of normoxia and hypoxia, respectively. These results indicate that the N203H mutation significantly affected HIF2-mediated transcription of *VEGF*, especially in hypoxic conditions (Figure 2).

### N203H mutation enhanced HIF2 and PHD2 protein interaction

**Figure 3:**
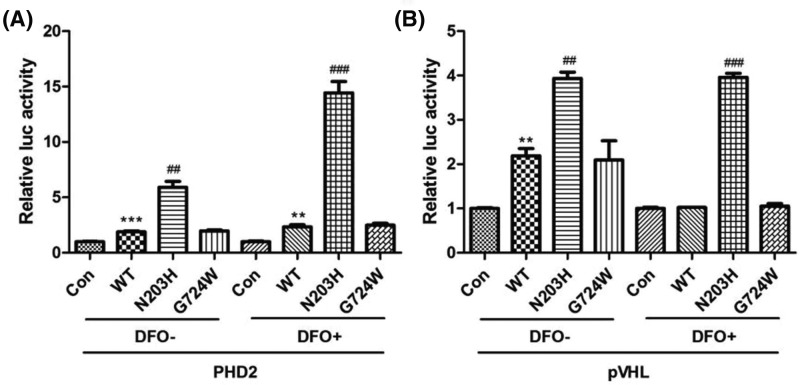
Effect of *EPAS1* mutations on HIF2–PHD2 and HIF2–pVHL interactions Mammalian two-hybrid system assays were conducted to evaluate protein–protein interactions. Cells were grown in conditions of normoxia (DFO-) or hypoxia (DFO+) for 24 h. The HIF2 N203H mutation showed enhanced HIF2 and PHD2 (**A**), and HIF2 and pVHL (**B**) protein–protein interactions, especially in conditions of hypoxia. **p<0.01; ***p<0.001 vs. empty vector; ##*p*<0.01; ###*p*<0.001 vs. wild-type (Student’s t-test).

A mammalian two-hybrid assay was used to evaluate whether *EPAS1* mutation altered the interaction between HIF2 and PHD2 (Figure 3A). After 24 h growth in normoxic or hypoxic conditions, cells transfected with the wild-type construct produced significantly more luciferase activity than did those transfected with the empty vector (***P<0.001 and **P<0.01). There was no difference in the luciferase activity of cells transfected with wild-type and G724W mutation constructs. Cells transfected with the N203H mutation construct showed a significant increase in luciferase activity compared with those transfected with the wild-type construct (^##^*P*<0.01 and ^###^*P*<0.001). The luciferase activity of cells transfected with the N203H mutation construct was approximately 3- and 6-fold greater than that of cells transfected with the wild-type construct in normoxic and hypoxic conditions, respectively. These results indicate that the N203H mutation enhanced protein–protein interactions between HIF2 and PHD2, especially in conditions of hypoxia.

### N203H mutation enhanced HIF2 and pVHL protein interaction

The mammalian two-hybrid assay was also used to evaluate whether the *EPAS1* mutation altered the interaction between HIF2 and pVHL (Figure 3B). After 24 h growth in normoxic conditions, cells transfected with the wild-type construct demonstrated significantly more luciferase activity than did those transfected with the empty vector (**P<0.01), while there was no difference between the two groups when grown in hypoxic conditions. No difference in luciferase activity was observed between cells transfected with wild-type and G724W mutation constructs. Cells transfected with the N203H mutant construct showed a significantly more luciferase activity than did those transfected with the wild-type construct (^##^*P*<0.01 and ^###^*P*<0.001). The luciferase activity of cells transfected with the N203H mutation construct was about one and a half- and four-fold greater than that of cells transfected with the wild-type construct in normoxic and hypoxic conditions, respectively. These results indicate that the N203H mutation enhanced protein–protein interactions between HIF2 and pVHL, especially in conditions of hypoxia.

## Discussion

We report the identification of two novel mutations (NM_001430: c.607A>C, p.N203H; c.2170G>T, p.G724W) in the *EPAS1* coding sequence in a group of Tibetan patients with non-syndromic CHD. Our functional experiments show that the N203H mutation, but not the G724W mutation, might significantly affect HIF2 transcriptional activity and protein stability. The results implicate *EPAS1* mutation might have a potential causative effect in the development of Tibetan non-syndromic CHD.

*EPAS1* is one of the most important plateau adaptive genes, especially in the Tibetan Plateau [14]. Mutations in the *EPAS1* coding sequence are associated with several human disorders, including altitude-related diseases [9–13,20,21]. Recently, *EPAS1* gain-of-function somatic mutations were reported in patients who presented with syndromic CHD [15]. Our previous study also indicated that CHD in Tibetan children might be associated with altitude levels and hypoxia [4]. Therefore, we speculated that *EPAS1* might be a candidate for Tibetan CHD. Sanger sequencing was performed in 286 non-syndromic Tibetan patients with CHD from Qinghai Province and we identified two novel *EPAS1* mutations. The two subjects carrying the mutations are Tibetan residents who had been diagnosed with PDA (N203H) and VSD (G724W), respectively. Both of the rare mutations identified here are predicted to be highly conservative and may affect protein structure and function.

*EPAS1* encodes HIF2α, which is involved in the regulation of chronic hypoxia stress [22]. Hypoxia is the most important HIF2α regulatory factor, and plays a role in the transcriptional activity and protein stability of HIF2α, and HIF2α expression is significantly increased after hypoxia treatment. As an important transcription factor, HIF2α has many target genes, including *VEGF* [23]. VEGF is an important angiogenic factor, and its function is essential for embryonic vasculogenesis and heart development [14,24]. Our results indicate that the HIF2α N203H mutation significantly affects HIF2 transcription activity at the *VEGF* promoter, especially in conditions of hypoxia. RT-qPCR confirmed that the identified mutations did not affect *EPAS1* mRNA expression, indicating that the mutations may affect HIF2α protein stability.

The hypoxic stress regulation of HIF2α mainly depends on its hydroxylation [25]. In normoxia, HIF2α is hydroxylated by PHD2. Hydroxylated HIF2α then promotes the binding of HIF2α to pVHL, the substrate receptor for an E3 ubiquitin ligase complex, leading to HIF2α protein degradation [6,7]. Therefore, in normoxia, the intracellular HIF2α protein content is very low. When the cells are in hypoxia, proline hydroxylase is inactivated and HIF2α cannot be hydroxylated or recognized by the VHL ubiquitin–proteinase complex [8]. Therefore, mammalian two-hybrid assays were performed to detect protein–protein interactions between HIF2 and PHD2, and between HIF2 and pVHL. Our results indicate that the N203H mutation enhanced protein–protein interactions between HIF2 and PHD2, especially in conditions of hypoxia. Therefore, the N203H mutation might promote the binding of HIF2α to pVHL and lead to HIF2α degradation. Our results confirmed that the N203H mutation significantly increased protein–protein interactions between HIF2 and pVHL. Taken together, our results suggested that the N203H mutation might affect HIF2 stability and *VEGF* transcription. *EPAS1* mutations might lead to Tibetan non-syndromic CHD. However, these results need to be confirmed with a larger sample size, and the mechanisms linking *EPAS1* mutations and Tibetan non-syndromic CHD require further elucidation.

## Supporting information

**Table S2:** Primers for Sanger sequencing

**Table S3:** Primers for plasmids construction and RT-qPCR

## Abbreviations

CHD: congenital heart disease
HIF: hypoxia-inducible factor
PDA: patent ductus arteriosus
PHD2: prolyl-hydroxylase domain 2
VEGF: ascular endothelial growth factor
VHL: von Hippel–Lindau tumor suppressor
VSD: ventricular septal defect

## References

[1] van der Linde D., Konings E.E., Slager M.A., Witsenburg M., Helbing W.A., Takkenberg J.J. (2011) Birth prevalence of congenital heart disease worldwide: a systematic review and meta-analysis. J. Am. College Cardiol. 58, 2241–2247 10.1016/j.jacc.2011.08.025 22078432

[2] Jin S.C., Homsy J., Zaidi S., Lu Q., Morton S., DePalma S.R. (2017) Contribution of rare inherited and de novo variants in 2,871 congenital heart disease probands. Nat. Genet. 49, 1593–1601 10.1038/ng.3970 28991257 PMC5675000

[3] Bigham A.W. and Lee F.S. (2014) Human high-altitude adaptation: forward genetics meets the HIF pathway. Genes Dev. 28, 2189–2204 10.1101/gad.250167.114 25319824 PMC4201282

[4] Chen Q.H., Wang X.Q. and Qi S.G. (2008) Cross-sectional study of congenital heart disease among Tibetan children aged from 4 to 18 years at different altitudes in Qinghai Province. Chin. Med. J. 121, 2469–2472 19187580

[5] Giaccia A.J., Simon M.C. and Johnson R. (2004) The biology of hypoxia: the role of oxygen sensing in development, normal function, and disease. Genes Dev. 18, 2183–2194 10.1101/gad.1243304 15371333 PMC517513

[6] Jaakkola P., Mole D.R., Tian Y.M., Wilson M.I., Gielbert J., Gaskell S.J. (2001) Targeting of HIF-alpha to the von Hippel-Lindau ubiquitylation complex by O2-regulated prolyl hydroxylation. Science 292, 468–472 10.1126/science.1059796 11292861

[7] Cockman M.E., Masson N., Mole D.R., Jaakkola P., Chang G.W., Clifford S.C. (2000) Hypoxia inducible factor-alpha binding and ubiquitylation by the von Hippel-Lindau tumor suppressor protein. J. Biol. Chem. 275, 25733–25741 10.1074/jbc.M002740200 10823831

[8] Lando D., Peet D.J., Whelan D.A., Gorman J.J. and Whitelaw M.L. (2002) Asparagine hydroxylation of the HIF transactivation domain a hypoxic switch. Science 295, 858–861 10.1126/science.1068592 11823643

[9] Percy M.J., Chung Y.J., Harrison C., Mercieca J., Hoffbrand A.V., Dinardo C.L. (2012) Two new mutations in the HIF2A gene associated with erythrocytosis. Am. J. Hematol. 87, 439–442 10.1002/ajh.23123 22367913 PMC3399664

[10] Percy M.J., Furlow P.W., Lucas G.S., Li X., Lappin T.R., McMullin M.F. (2008) A gain-of-function mutation in the HIF2A gene in familial erythrocytosis. N. Engl. J. Med. 358, 162–168 10.1056/NEJMoa073123 18184961 PMC2295209

[11] Perrotta S., Stiehl D.P., Punzo F., Scianguetta S., Borriello A., Bencivenga D. (2013) Congenital erythrocytosis associated with gain-of-function HIF2A gene mutations and erythropoietin levels in the normal range. Haematologica 98, 1624–1632 10.3324/haematol.2013.088369 23716564 PMC3789469

[12] van Wijk R., Sutherland S., Van Wesel A.C., Huizinga E.G., Percy M.J., Bierings M. (2010) Erythrocytosis associated with a novel missense mutation in the HIF2A gene. Haematologica 95, 829–832 10.3324/haematol.2009.017582 20007141 PMC2864390

[13] Lorenzo F.R., Yang C., Ng Tang Fui M., Vankayalapati H., Zhuang Z., Huynh T. (2013) A novel EPAS1/HIF2A germline mutation in a congenital polycythemia with paraganglioma. J. Mol. Med. 91, 507–512 10.1007/s00109-012-0967-z 23090011 PMC3570726

[14] Beall C.M., Cavalleri G.L., Deng L., Elston R.C., Gao Y., Knight J. (2010) Natural selection on EPAS1 (HIF2alpha) associated with low hemoglobin concentration in Tibetan highlanders. Proc. Natl. Acad. Sci. U.S.A. 107, 11459–11464 10.1073/pnas.1002443107 20534544 PMC2895075

[15] Vaidya A., Flores S.K., Cheng Z.M., Nicolas M., Deng Y., Opotowsky A.R. (2018) EPAS1 mutations and paragangliomas in cyanotic congenital heart disease. N. Engl. J. Med. 378, 1259–1261 10.1056/NEJMc1716652 29601261 PMC5972530

[16] Takeda N., Maemura K., Imai Y., Harada T., Kawanami D., Nojiri T. (2004) Endothelial PAS domain protein 1 gene promotes angiogenesis through the transactivation of both vascular endothelial growth factor and its receptor, Flt-1. Circ. Res. 95, 146–153 10.1161/01.RES.0000134920.10128.b4 15192019

[17] Chang C.P., Neilson J.R., Bayle J.H., Gestwicki J.E., Kuo A., Stankunas K. (2004) A field of myocardial-endocardial NFAT signaling underlies heart valve morphogenesis. Cell 118, 649–663 10.1016/j.cell.2004.08.010 15339668

[18] Ema M., Taya S., Yokotani N., Sogawa K., Matsuda Y. and Fujii-Kuriyama Y. (1997) A novel bHLH-PAS factor with close sequence similarity to hypoxia-inducible factor 1alpha regulates the VEGF expression and is potentially involved in lung and vascular development. Proc. Natl. Acad. Sci. U.S.A. 94, 4273–4278 10.1073/pnas.94.9.4273 9113979 PMC20712

[19] Woo K.J., Lee T.J., Park J.W. and Kwon T.K. (2006) Desferrioxamine, an iron chelator, enhances HIF-1alpha accumulation via cyclooxygenase-2 signaling pathway. Biochem. Biophys. Res. Commun. 343, 8–14 10.1016/j.bbrc.2006.02.116 16527254

[20] Yang C., Sun M.G., Matro J., Huynh T.T., Rahimpour S., Prchal J.T. (2013) Novel HIF2A mutations disrupt oxygen sensing, leading to polycythemia, paragangliomas, and somatostatinomas. Blood 121, 2563–2566 10.1182/blood-2012-10-460972 23361906 PMC3612863

[21] Brusselmans K., Compernolle V., Tjwa M., Wiesener M.S., Maxwell P.H., Collen D. (2003) Heterozygous deficiency of hypoxia-inducible factor-2alpha protects mice against pulmonary hypertension and right ventricular dysfunction during prolonged hypoxia. J. Clin. Invest. 111, 1519–1527 10.1172/JCI15496 12750401 PMC155039

[22] Sharma A.K. and Khanna D. (2013) Diabetes mellitus associated cardiovascular signalling alteration: a need for the revisit. Cell. Signal. 25, 1149–1155 10.1016/j.cellsig.2013.01.022 23385086

[23] Boddy J.L., Fox S.B., Han C., Campo L., Turley H., Kanga S. (2005) The androgen receptor is significantly associated with vascular endothelial growth factor and hypoxia sensing via hypoxia-inducible factors HIF-1a, HIF-2a, and the prolyl hydroxylases in human prostate cancer. Clin. Cancer Res.: An Off. J. Am. Assoc. Cancer Res. 11, 7658–7663 10.1158/1078-0432.CCR-05-0460 16278385

[24] Ferrara N., Carver-Moore K., Chen H., Dowd M., Lu L., O’Shea K.S. (1996) Heterozygous embryonic lethality induced by targeted inactivation of the VEGF gene. Nature 380, 439–442 10.1038/380439a0 8602242

[25] Kaelin W.G. Jr and Ratcliffe P.J. (2008) Oxygen sensing by metazoans: the central role of the HIF hydroxylase pathway. Mol. Cell 30, 393–402 10.1016/j.molcel.2008.04.009 18498744

