# Supplementary material for: Mutations in EPAS1 in congenital heart disease in Tibetans
Source: Biosci Rep. 2018 Dec 18;38(6):BSR20181389. doi: 10.1042/BSR20181389 (PMC6435565; doi:10.1042/BSR20181389)
Supplement: Supplementary file 1 [file bsr20181389_Supp1.pdf]

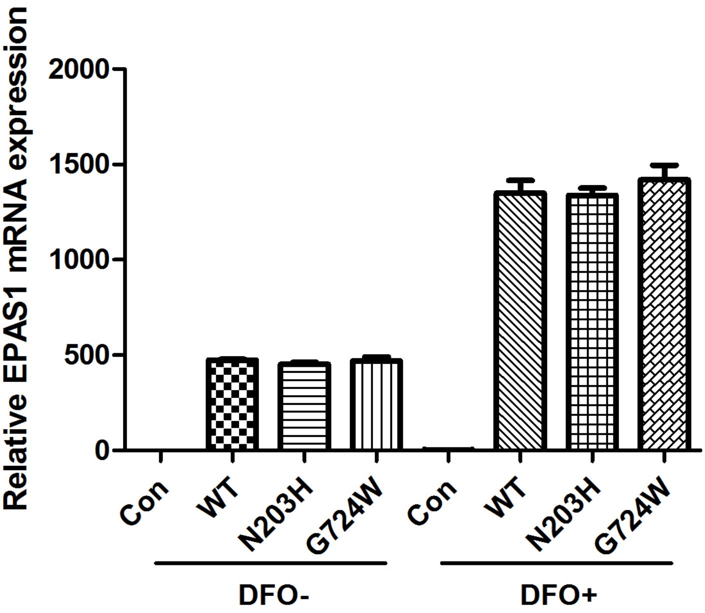

Relative EPAS1 mRNA expression
2000
1500
1000
500
0
Con
WT
N203H
G724W
Con
WT
N203H
G724W
DFO-
DFO+

Supplementary File

Table S2. Primers for Sanger sequencing

ID	Sequences(5'--3')	Length(bp)
EPAS1-1	TTCGGGTCTGACAGCCTC	599
	CTGGGAGATTTTCAAAGGCAC	
EPAS1-2	CACAGGGAATGCAAGAGGAG	611
	GTAAAGGTCACTGCTGGAAGAG	
EPAS1-3	GGTGTCCTCTGTGCAGTG	446
	CATTTGCAGGGAGAGGTC	
EPAS1-4	GACCTGACACTGGACTGGGAT	559
	GTGGTGTCATTTGGCAGAGAT	
EPAS1-5+6	GCACCACTGACCATGTTC	886
	CAATGAAACAGTGGCACAGGT	
EPAS1-7	CCTTCTGGGGTTAGCTCCT	365
	GTGGCAGCCTTGGTTTTCT	
EPAS1-8	CCTGTTCTGTCTGTTCCCCT	332
	TCCTGAGTCTGGGAAGCTTG	
EPAS1-9	TCCGAAACAGACATACATT	861
	TCCCAACATTACTCCAAC	
EPAS1-10	TTTTAGATGGGTTCTACAGC	970
	TGAGGACCAAACAGGAT	
EPAS1-11	GGCTGAAGGGACATTTG	791
	TGACCACAGGCTCCATT	
EPAS1-12	TGAGATGAATGGCTCTGC	749
	CACATGGCTTGAGGTGAT	
EPAS1-13	CACAGGCATCAGCATTG	553
	CTTCATCCGTTTCCACATC	
EPAS1-14	GCTCCAGACTCCCTCATAG	756
	GCCAACCTTCAGGACC	
EPAS1-15	CTGAGCCTTGTTAGAATGG	689
	TTGGGCAGTCGGTAGT	
EPAS1-16	AGGCAAGAAGAAAGGACTG	1076
	GGAGACGCCACAAACC	

Supplementary File

Table S3. Primers for plasmids construction and RT-qPCR

**ID**	**Sequences(5’--3’)**
pCDNA3.1(+)-EPAS1	CCCAAGCTTATGACAGCTGACAAGGAGAAGAAAAG
	CGCGGATCCTCAGGTGGCCTGGTCCAGGGCT
EPAS1 A607C	GGTGAAAGTCTACCACAACTGCCCTCC
	GGAGGGCAGTTGTGGTAGACTTTCACC
EPAS1 G2170T	AGGACCTGAGCTGGGGGGACCCACCT
	AGGTGGGTCCCCCCAGCTCAGGTCCT
pGL3basic-VEGF promoter	CCTCGAGGAAGATGAGCTATGAGTCTGGGCT
	CAAGCTTCTGCCCaCTGGTCTCTGGCT
PFN11A(BIND)-EPAS1	TTCTGCGATCGCCATGACAGCTGACAAGGAGAAGAAA AGGAGT
	GGGGGTTTAAACGGTGGCCTGGTCCAGGGCTCT
PEN10A(ACT)-VHL	TAAAGCGATCGCCATGCCCCGGAGGGCGGAGAA
	GTCGGTTTAAACATCTCCCATCCGTTGATGTGCAATGCG
PEN10A(ACT)-PHD2	TAAAGCGATCGCCATGGCCAATGACAGCGGCG
	AACTGTTTAAACGAAGACGTCTTTACCGACCGAATCT
EPAS1-qPCR	ACGCCACCCAGTACCAGGA
	AATGAGGGCCCGAGCAGC
β-actin-qPCR	CTCCATCCTGGCCTCGCTGT
	ACTAAGTCATAGTCCGCCTAGA
